# Body image is associated with persistence. A study of the role of weight-related stigma

**DOI:** 10.3389/fpsyt.2024.1464939

**Published:** 2024-10-25

**Authors:** Wojciech Styk, Ewa Wojtowicz, Paweł Glibowski, Katarzyna Iłowiecka, Aleksanda Jędryszek-Geisler, Szymon Zmorzyński

**Affiliations:** ^1^ Academic Laboratory of Psychological Tests, Medical University of Lublin, Lublin, Poland; ^2^ Chair of Pedeutology and Psychology of Education, Christian Theological Academy of Warsaw, Warsaw, Poland; ^3^ Department of Biotechnology, Microbiology and Human Nutrition, Faculty of Food Sciences and Biotechnology, University of Life Sciences in Lublin, Lublin, Poland; ^4^ Nutrition Clinic, Department of Clinical Dietetics Medical University of Lublin, Lublin, Poland; ^5^ Department of Psychology, Institute of Pedagogy and Psychology, Management Academy of Applied Sciences in Warsaw, Warsaw, Poland; ^6^ Laboratory of Genetics, Academy of Zamość, Zamość, Poland

**Keywords:** persistence, body image, body mass index, weight stigma, stress, cortisol, dehydroepiandrosterone, temperament

## Abstract

**Abstract:**

The study replicates a preliminary report from 2019 on therelationship between body image and persistence.

**Purpose:**

The aim of our study was to analyze the associations between body image, persistence, and body weight stereotypes.

**Patients and methods:**

A total of 750 individuals were recruited for the study. The research was carried out in computer labs. The procedure consisted of psychological questionnaires (Persistence Scale, The Body Esteem Scale, Perceived Weight Stigma Questionnaire, Weight Bias Internalization Scale, Hospital Anxiety and Depression Scale, Formal Characteristics of Behavior – Temperament Inventory, and NEO-PI-R) and The Maze Test (a computer tool). After completing the Simple Maze Test, saliva samples were collected. Next, the subjects proceeded to the laboratory where anthropometric and body composition measurements were taken. The hormone levels (cortisol and dehydroepiandrosterone) in the collected saliva samples were analyzed via ELISA to determine stress.

**Results:**

Body image and persistence are related variables. They are associated with the internalization of stereotypes and perceived stigma related to body weight. These associations are differentially shaped according to sex and the regularity of body weight. In women, a stronger association of these variables with body image was observed, while in men, the relationship with body image was weaker, with a stronger association shown by perceived weight-related stigma. In the group of participants with a BMI<18.5, there was no significant association between the internalization of stereotypes and the analyzed variables. This relationship appeared in the group of subjects with a normal body weight and was strongest in the group of participants who were overweight or obese. Perceived weight-related stigma was most strongly associated with body image in the group with BMI<18.5 kg/m2 and with persistence in the group with BMI>25 kg/m2.

**Conclusion:**

Body-related stigma affects not only overweight and obese individuals and its mechanisms may be shaped differently.

## Introduction

In recent years, there has been a significant increase in interest in obesity. This results from the critical increase in weight among the population. According to the WHO, in 2022, 2.5 billion adults over the age of 18 were overweight, and more than 890 million adults suffered from obesity (1). Researchers in various disciplines are constantly searching for risk factors and causes of obesity. Moreover, they have analyzed the costs and consequences of excessive body weight and related phenomena as well as constructed practical solutions to limit the physical, mental, and social effects of obesity.

Currently, obesity is known to be a multifactorial disease. The main causes of this disorder are the mutual, multilevel interrelationships between genetic, physiological, and environmental factors (2–5). The consumption of foods whose energy value exceeds the body’s energy expenditure is considered the primary mechanism responsible for the development of obesity. This approach, although correct, does not fully explain the phenomenon of the worldwide trend of weight gain.

The human body tolerates weight gain more easily than weight loss, making it relatively simple to consume more calories than a person needs (6). Today, in a world with easy access to food, eating behavior is motivated not only by hunger but also by the satisfaction of eating (7). Studies show that reward sensitivity affects diet—the greater it is, the greater the consumption of fast food and sugar-rich foods (8). Self-control and persistence can be helpful in resisting the temptation of a high-energy and unhealthy diet by constructing and maintaining healthy eating habits. Persistence, according to FrazierKliknij tutaj, aby wprowadzić tekst (9)., is a trait that is positively associated with health behaviors with the goal of preventing diseases that are common causes of death (e.g., cardiovascular diseases, stroke). People with obesity are at risk of premature deaths associated with being overweight and having comorbidities. Persistence may, therefore, be one of the protective factors against the negative effects of obesity.

People struggling with excess body weight may have a reduced ability to self-regulate. A lower BMI is linked to greater persistence in achieving long-term goals. Compared with normal-weight individuals, obese people may have a weaker ability to delay gratification, which translates into persistence in maintaining proper diets (10). This was also the case for the Styk study (11); reinforcing effort can be a protective factor in maintaining self-control. Low persistence in obese and overweight people is mainly related to difficulties in stopping irrelevant thoughts or memories (12). Other studies, in turn, found that regardless of the normality of one’s weight, people who rated their weight as too high had lower persistence rates than those who perceived their weight as normal (13). The importance of persistence in preventing obesity, especially among children and adolescents, is also highlighted by Yu et al. (14), who found that schools should focus on increasing persistence and promoting a positive body image to prevent obesity in children and adolescents.

In the literature concerning obesity, much attention has been given to the issue of body image, which is defined as a way of perceiving one’s own body that takes into account related thoughts and feelings. Body image is a multidimensional construct and can be positive or negative. On the one hand, people adequately evaluate their body and are satisfied with it. On the other hand, negative body image is associated with inaccurate perceptions and critical evaluation of the body (15, 16). Research conducted by Aime (17) in eight countries and seven different languages showed some similarities in terms of assessing the various indicators that make up the self-image construct, which was explained by globalization processes. However, Aime et al. noted that measures of self-image are sensitive to cultural context. A negative body image can cause inappropriate eating behavior (18) and limit the possibility of weight loss. In contrast, a positive body image may be a predictor of maintaining health habits even two years after bariatric surgery (19). Numerous studies indicate that many obese individuals show dissatisfaction with their bodies, which negatively affects quality of life and functioning in various spheres. According to Troisi (15), women are more dissatisfied with their bodies than men compared to their normal-weight peers.

The suffering of individuals struggling with excessive body weight is exacerbated by the supremacy of the slim beauty ideal promoted by most cultures. Sociocultural factors determine how people view their appearance. Western culture in particular makes beauty dependent on low body weight, which negatively affects the mental health of people with obesity, although the relationship is not conclusive (20). Appearance pressure primarily affects women, resulting in a lack of satisfaction with their appearance (21), while research by Silva (22) has shown that young women who are subjected to environmental pressures internalize a slim figure, which negatively affects their satisfaction with their bodies. Unattainable canons of beauty are promoted primarily by the media, and comparison with an ideal model promotes low evaluation of one’s own body. The use of appearance-focused media adversely affects self-image whereby a positive self-image is associated with the capacity to engage in healthy behaviors and resist unrealistic appearance canons promoted by the media (23).

Widespread stereotypes about people with obesity contribute to the prevalence of negative physical and mental health phenomena. The literature indicates that people with excessive body weight may experience, among other things, stress, mood disorders, decreased self-esteem and self-worth [especially among women (24)], or various eating disorders (25–28). Re-education on weight-related stigma may predict improvements in mental health (29).

Body weight can cause discrimination in various aspects of life; it is common in work, education, health care, sports, or interpersonal relationships (30–36). In the case of obese people, the body becomes a key criterion for judging them against social norms constructed for a particular trait. Thus, the difference between expectations and actual body weight determines who will be stigmatized and how they will cope with devaluation and lower status (34). At the same time, oppressive attitudes toward obese people seem to be more socially acceptable than other forms of prejudice (37) and discrimination based on body weight. It is socially tolerated, even though research shows that it may increase the risk of obesity (38–41).

The internalization of socially defined, negative attitudes about the body also contributes to self-devaluation and lower self-esteem (35, 42, 43). People with internalized weight stigma tend to evaluate themselves negatively and expect others to stigmatize them because of this aspect of their appearance (44). Consequently, they develop difficulties in regulating their weight, which increases overeating and body image deterioration (45). A protective factor against the impact of prejudice on self-esteem may be the awareness of being a target of prejudice; rejection of discrimination may be associated with less suffering (46). Haga (47) showed that people with obesity use a variety of defense strategies to cope with negative social reactions and judgments about their body weight.

Stigmatization due to excessive body weight is a common phenomenon (48). The main feature of these biases is the perceived ability to control weight (49). Overweight people are perceived as lazy, passive, lacking willpower and control, and unattractive (45, 50). The stereotype of obesity, leading to prejudice and discrimination, assumes a lack of persistence in building healthy habits and taking care of one’s figure (e.g., ‘just eat less and exercise more’). Responsibility for being overweight or obese is attributed only to overweight individuals, even though the mechanisms involved are complex (51).

### The current study

A preliminary report on the relationship between body image and persistence was published in 2019 (13). The results of this study showed, among other findings, a relationship between persistence and subjective body weight. Significantly, lower rates of persistence were achieved by those assessing their body weight as too high, although their BMI identified their body weight as normal.

The aim of this project was to replicate the 2019 study and reduce its limitations by doing the following:

1. Increasing the study group and restricting the age of the study participants to 30 years to exclude the influence of cognitive decline with age on The Maze Test results.
2. Conducting The Maze Test under laboratory conditions (not via the website as previously described).
3. Introducing anthropometric measurements and body composition to better determine the correctness of one’s body weight.
4. Extending the research because The Maze Test tool only measures persistence from a situational perspective, therefore, variables that could modify the obtained results were included, such as temperament, personality, levels of depressive symptoms, and stress levels.
5. Including persistence as a trait in the research.
6. Including the variables related to weight stereotypes.

In this study, we posited the following hypotheses: (I) body image is related to persistence taken from both situational and trait perspectives; (II) persistence and body image are associated with body weight stereotypes; and (III) persistence is related to temperamental-personality traits, but the relationship of persistence with body image is significant.

## Materials and methods

### Participants

This study was carried out as part of the Ministry of Science and Higher Education (Poland) grant ‘Science for Society’. As part of the grant, educational activities were planned in the form of health information meetings focused on topics such as proper nutrition, the impact of stress on health, and the importance of body weight in the development of metabolic diseases. During these meetings as well as via dedicated posters and posts on social media, the study participants were recruited. Each individual was informed about the study inclusions/exclusions (inclusion: up to 30 years of age, exclusions: mental disorders, pregnancy, pacemaker, metal prostheses), the purpose of the study, the time needed to complete it, and the procedures to be carried out. Applications for the study were accepted by email and via a Google Forms questionnaire. To take part in the study, participants received a reward in the form of a voucher worth PLN 100 (approximately €23) to be used at a popular sales site. Participants also underwent a body composition analysis via consultation. The participants who were particularly interested were offered psychodietetic consultations.

A total of 750 people were recruited for the study (during information meetings or via social media). After a review of the collected data, the data of 721 individuals were included in the analysis. The exclusion of 23 people from the analysis was due to high levels of depressive/anxiety symptoms, and 6 people were excluded due to missing data (incorrect or incomplete psychological questionnaires, including resignation during the study). In the recruited group, 56% of individuals were working (of whom 35% were working and studying), 43% were only studying, and the rest indicated that they were not studying or working. Most participants (N=395) had a BMI in the normal range. Another large group (N=228) was made up of individuals with a higher BMI (indicating overweight and obesity). The smallest group included individuals (N=98) with a BMI below normal range.

A description of the study group is shown in **Table 1**.

**Table 1:** Characteristics of the study participants.

	BMI		Age		Body fat (%)		Body mass	
	Female	Male	Female	Male	Female	Male	Female	Male
Valid	584	137	584	137	584	137	584	137
Mean	22.88	25.80	21.90	21.51	27.77	22.40	62.46	83.76
Std. Deviation	4.67	4.44	4.16	2.74	6.87	6.14	13.53	15.99
Minimum	14.80	15.80	18.00	18.00	8.00	3.70	37.70	48.20
Maximum	42.20	43.80	56.00	40.00	47.80	36.60	125.70	140.20

### Procedure

**Figure 1:**
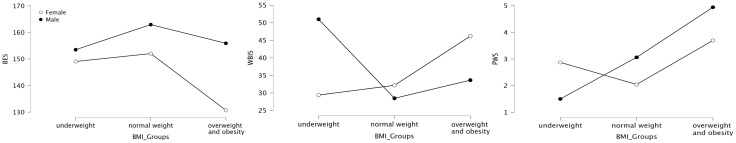
The procedure used to conduct the study.

The surveys started between 8 and 11 am and were conducted in rounds of up to 20 participants. The research was carried out in computer laboratories at the universities involved in the project. When the participants arrived, they were informed about the purpose of the study, the exclusion criteria and the procedures to be followed in the study. In the next step, the subjects signed an informed consent form to participate in the study. Informed consent was obtained at the start of the procedure, which consisted of handing over the psychological questionnaire forms and saliva (at least 0.5 ml) sampling (into 1.5 ml laboratory tubes). The researcher displayed a presentation to the participants with the steps to be followed. The entire procedure is shown in **Figure 1**. The procedure was conducted so that the start of the Maze Test began at the same time for all participants in a given test round so that individuals were not distracted by their surroundings. After completing the Maze Test and collecting the third saliva sample (as previously described), the subjects returned to the laboratory where anthropometric and body composition measurements were taken. The collected saliva samples were immediately frozen at -20°C and transferred to the laboratory, where hormone (cortisol, dehydroepiandrosterone - DHEA) levels were determined. The hormone levels were analyzed only in saliva samples collected after the Maze Test.

### Measures

#### Body mass index

**Figure 2:**
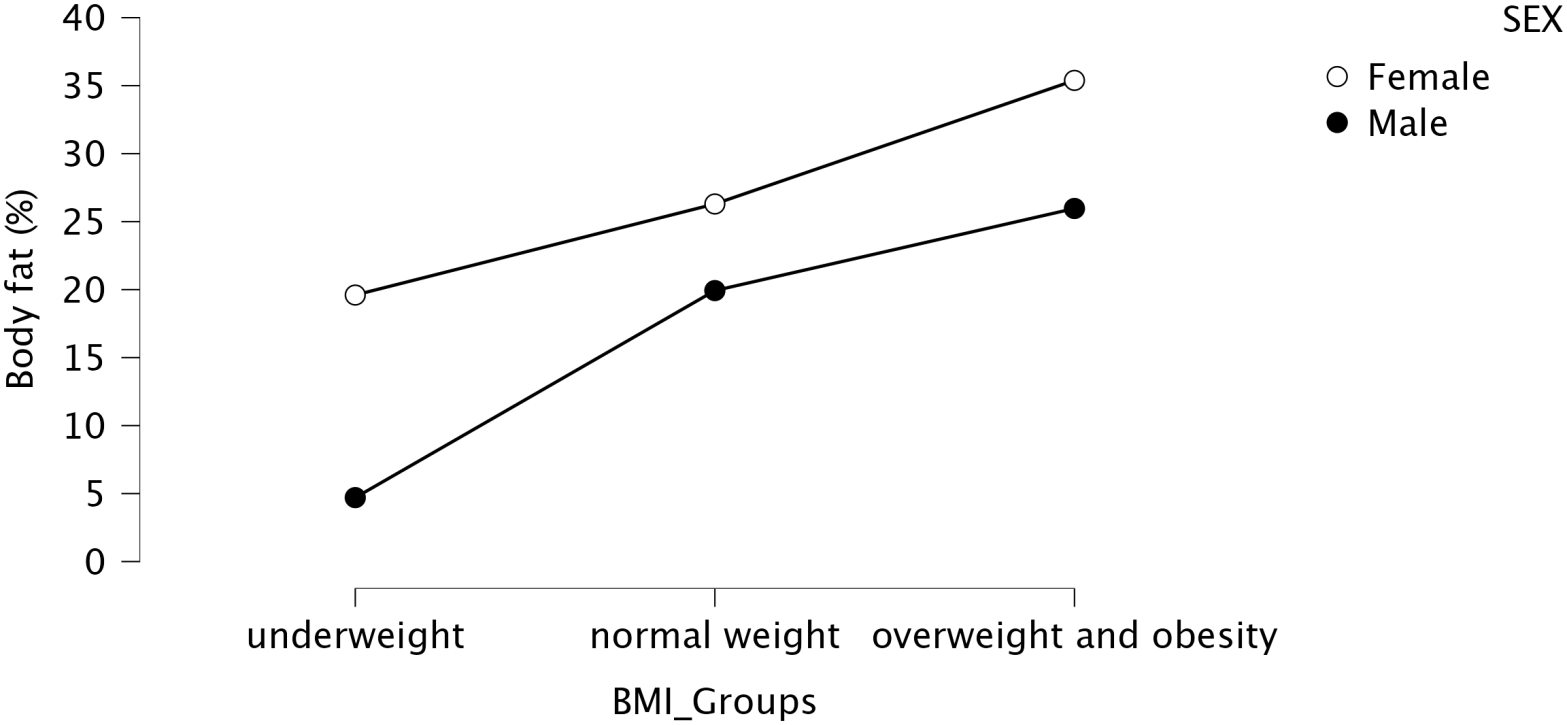
Relationship of mean body fat percentage to BMI according to sex.

To determine the correctness of one’s body weight, the BMI was used. Due to doubts that appear in the literature regarding this index, confirmatory measurements of body composition were also taken. Body weight and body composition were determined using a TANITA MC-780 P MA multifrequency, segmented body composition analyzer. Body height was determined using a Leicester Tanita HR 001 growth meter. BMI was calculated according to general guidelines, and the correctness of body weight was confirmed by body composition analysis. This finding was confirmed by the strong correlation between body fat percentage and BMI (r=0.73; p<0.01). **Figure 2** shows the correlation of the mean body fat percentage with BMI according to the subjects’ sex.

#### Persistence

The measurement of persistence was planned from a trait perspective (persistence) and a situational perspective. The persistent scale (PS-20) was used to measure persistence as a trait. This scale was developed on the concept of persistence as a resource, a positive factor related to individual adaptive behavior, mental toughness, and appropriate self-regulation. The scale was constructed as a 20-item self-report tool. The authors confirmed its accuracy and high reliability (Cronbach’s alpha = 0.97) (11). The reliability obtained in our study was 0.96. In contrast, persistence from a situational perspective was measured using a computerized tool called the Maze Test. This tool was used in the original study (52). The properties of the tool have also been confirmed in subsequent studies (13, 53). The Simple Maze Test was constructed as a computer-based task in which the examinee is given simple maze tasks to solve. Unlike the original tool, this study abandoned the web-based tool in favor of a software tool installed on a computer running the Windows operating system. In addition, in this study, participants were given trial tasks before solving the assessed task. In this way, the subjects learned how to complete the tasks. The individuals performed the tasks on computers equipped with mice and 17-inch screens. The assessment task consisted of solving as many maze tasks as possible. Once the task was solved correctly, the next maze was displayed until the test subject gave up and clicked the ‘abort game’ button. Two indicators were used to determine the subjects’ situational persistence: (I) number of tasks performed (NoTasks), which is an indicator that determines persistence, taking into account only the efficiency of solving tasks; and (II) time spent on tasks (Time), which is an indicator that determines persistence without taking into account effectiveness, that is, only the time spent on the activity.

#### Body image

To determine satisfaction with different aspects of the body, the Body Esteem Scale (BES) developed by Franzoi and Shields was used (54). This study used the Polish adaptation by Lipowska and Lipowski (55). The scale includes body-related aspects (e.g., smell) and body parts (e.g., nose, thighs). The subjects responded on a 5-point scale from strongly dislike to strongly like regarding their attitudes toward the listed aspects and body parts. The scale provides an overall score representing respondents’ attitudes toward their bodies. The higher the score is, the more positive the attitude toward the body. The reliability obtained in our study was 0.71.

#### Perceived weight stigma

We used the Perceived Weight Stigma Questionnaire (PWS) to determine an individual’s level of perceived weight stigma. This scale consists of 10 items scored dichotomously (a score of 0 means “no”, and a score of 1 means “yes”). An example item is “Because of your weight you are treated with less respect than others”. PWS is a unidimensional factor structure with satisfactory fit indices, as demonstrated by confirmatory factor analysis (56). The responses were summed, with higher scores indicating greater perceived weight stigma. Research on PWS in different cultures has confirmed its good performance (56, 57). For study purposes, the scale was translated into Polish by a team of competent judges. Analysis of the reliability of the results obtained for the Polish sample showed satisfactory values for the scale (Cronbach’s alpha was 0.76).The Polish version of the scale is included in the **Supplementary Material**.

#### Internalization of weight bias

To measure internalized stereotypes related to body weight, the modified weight bias internalization scale (WBIS) was used. The scale consists of 11 items, and responses are given on a 7-point Likert scale (from ‘strongly disagree’ to ‘strongly agree’) assessing the internalization of weight-related attitudes or self-directed stigma. An example item from the scale is “Because of my weight, I am less attractive than most other people”. The results indicated that the WBIS has high internal consistency and strong construct validity (58, 59). For the purposes of this study, the scale was translated into Polish by a team of competent judges. Analysis of the reliability of the results obtained for the Polish sample showed satisfactory values for the scale (Cronbach’s alpha was 0.80). The Polish version of the scale is included in the **Supplementary Material**.

#### Depression, anxiety, anger

The levels of depressive symptoms, anxiety, and anger were determined using the Hospital Anxiety and Depression Scale (HADS) developed by Zigmond and Snaith (60). This screening tool is popular in both clinical practice and research. It contains three scales that correspond to levels of general anxiety (HADS_A), depression (HADS_D), and anger (HADS_Ag). Our study used the Polish version, which has satisfactory psychometric properties (61). In our study reliability was measured by Cronbah’s alpha. It obtained satisfactory values and was as follows: HADS_A – 0.68; HADS_D – 0.70; HADS_Ag - 0.65

#### Stress and ELISA methods

The DHEA/cortisol ratio has been used as a biomarker of chronic stress, with DHEA acting to offset the effects of cortisol. This ratio is one of several physiological biomarkers that have been identified for chronic stress (62). Cortisol levels were used as a biomarker of acute stress. The measurement of cortisol and DHEA has been identified as a promising approach for monitoring stress and assessing mental health (63, 64).

Hormone concentrations were determined in saliva samples using an enzyme-linked immunosorbent assay (ELISA). Specific ELISA kits (Demeditec, Kiel, Germany) were used (according to the manufacturer’s protocol) to determine the levels of testosterone, DHEA, and cortisol in the saliva samples. A Multiskan FC plate reader (Thermo Scientific, Waltham, MA, USA) at a wavelength of 450 nm was used for the measurement of hormone levels. Each participant’s samples were analyzed in two replicates to reduce error variance. The mean cortisol level among the subjects was 15.89 ng/ml and ranged from 0.50 to 30.0 ng/ml. The mean DHEA concentration among the individuals was 623.02 pg/ml, ranging from 4.18 to 2705.00 pg/ml. To facilitate data comparison and reduce sensitivity to outlier observations, an index of acute and chronic stress was created by ranking cortisol levels and cortisol-DHEA ratios, respectively. The indices thus created were used in the analyses as chronic stress (chronic stress) and acute stress (acute stress) indices.

#### Temperament

The Polish revised version of the Formal Characteristics of Behavior - Temperament Inventory (FCZKT) by M. Cyniak-Cieciura, J. Strelau, and B. Zawadzki was used to determine the temperamental traits of the subjects. This scale is based on the Regulatory Theory of Temperament, developed by Jan Strelau, and describes human behavior in seven temperamental traits: Rhythmicity briskness (FCZKT_BR), perseveration (FCZKT_PE), sensory sensitivity (FCZKT_SS), endurance (FCZKT_EN), emotional reactivity (FCZKT_ER), and activity (FCZKT_AC). This method makes it possible to diagnose the basic dimensions of temperament, which are biologically determined. The questionnaire consists of 100 statements to which the subject must respond on a 4-point scale (1 - strongly disagree; 2 - disagree; 3 - agree; 4 - strongly agree). The scale has satisfactory psychometric properties. In our study reliability was measured by Cronbah’s alpha. It obtained satisfactory values and was in the range of 0.69-0.73.

#### Personality

The subjects’ personality traits were determined based on a five-factor model using the NEO-PI-R personality inventory developed by Costa and McCrae (65). According to the assumptions of this model, human personality can be described along five dimensions: neuroticism (NEO_N), extraversion (NEO_E), openness to experience (NEO_O), conscientiousness (NEO_C), and agreeableness (NEO_A). This study used the Polish abridged version of the inventory, which contains 60 items (66). In our study reliability was measured by Cronbah’s alpha. It obtained satisfactory values and was in the range of 0.72-0.83.

#### Statistics

JASP 0.18.3 software was used to carry out the statistical analyses. The collected data were first analyzed to determine the applicability of the parametric tests. The collected data met the criteria for the use of parametric tests. The r-Pearson test was applied to determine the strength of correlation. ANOVA was used for comparisons between the groups. Comparisons of correlation strength within the groups were made by Fisher’s Z test using the calculator available at www.psychometrica.de. To confirm the obtained results and graphically depict the relationships between the main variables in this study, a network analysis was conducted. This is a relatively new and promising method for modeling interactions between variables. The analysis is based on the concept of estimating a direct relationship between all variables rather than trying to reduce the structure of variables to their shared information. The analyses carried out are based on the bootstrap R package (67). The network graphs produced by JASP 018.3 are based on the graph of the R package (68). The stability of each estimated network, including the accuracy of edge weights and centrality, was assessed. The accuracy of the edge weights was estimated by drawing nonparametric bootstrapped confidence intervals (CIs) with 1000 permutations. Narrow bootstrapped CIs indicated low sampling variability in edge weights, indicating an accurate network. Stability of strength was examined using bootstrapped subsets with dropped cases to assess how well the order of centrality was preserved in a portion of the data.

## Results

The analysis of the collected data began with determining whether there were differences according to sex and the normality of the subjects’ body weight (BMI_groups). The normality of body weight was estimated using BMI with the subjects divided into three groups: underweight, normal weight, and overweight or obese. Due to the volume of data, tables with descriptive statistics are included in the **Supplementary Material**, and the results of the analyses are presented below.

**Figure 3:**
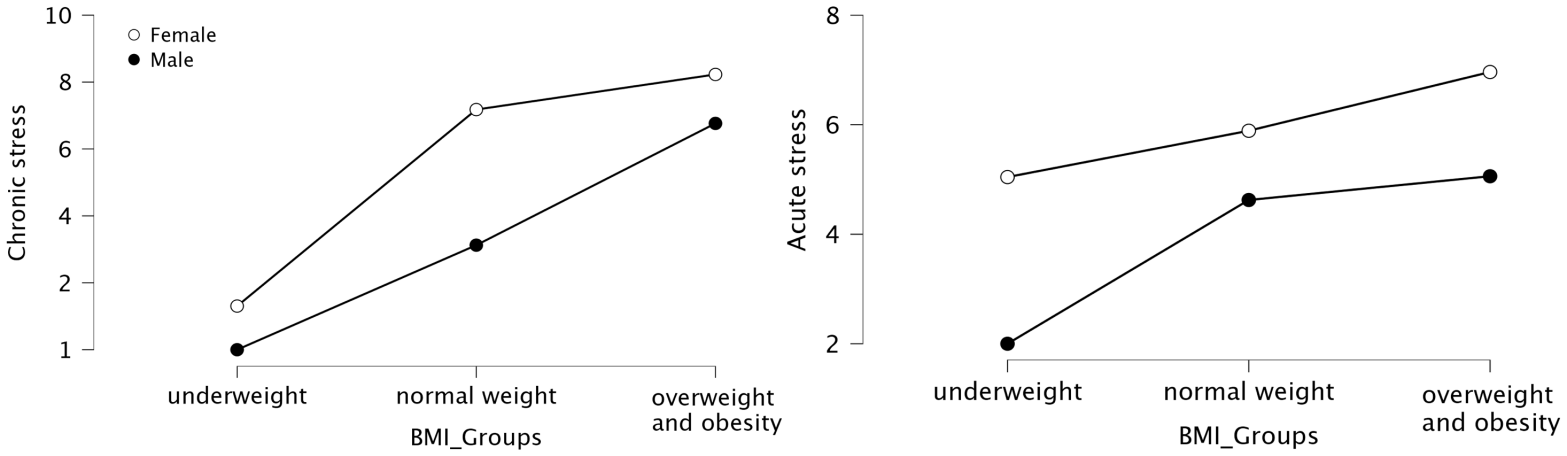
Comparison of the subjects’ persistence in the SEX and BMI groups.

The conducted analyses did not show that sex or BMI significantly differentiated the results of persistence as a trait and situational persistence indicator. The results of the analyses are shown in **Table 2** and **Figure 3**.

**Table 2:** Comparison of the respondents’ persistence in the groups according to SEX and BMI.

	Numberof tasks performed		Time spent on tasks		Persistence_Trait	
Cases	F	p	F	p	F	p
SEX	2.48	0.12	9.83	0.12	0.14	0.71
BMI_Groups	0.05	0.96	1.13	0.32	1.08	0.34
SEX ✻ BMI_Groups	0.40	0.67	1.54	0.22	0.09	0.92

**Figure 4:**
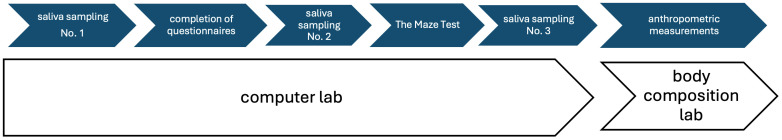
The graphical representation of the results comparing BES, WBIS, and PWS in the SEX and BMI groups.

Analysis of the mean values on the BES revealed differences according to sex and BMI group. The mean scores on the BES indicated that women rated their bodies lower than men (p=0.04). Differences due to BMI were found to be significant for the normal weight, overweight, and obese groups (p_tukey_<0.01). Overweight and obese respondents rated their bodies lower than those with normal body weight. As determined by the WBIS scale, the internalization of body weight stereotypes did not appear to differ according to sex. Differences were shown due to BMI groups and the interaction of sex and BMI groups. There were significant differences (p_tukey_<0.01) in the following comparisons: underweight females vs. overweight and obese females; normal weight females vs. overweight and obese females; normal weight males vs. overweight and obese females; and overweight and obese females vs. overweight and obese males. In contrast, the mean PWSs were differentiated only by BMI group. On average, overweight and obese individuals scored higher on the PWS scale than normal-weight subjects (p_tukey_<0.01). The results of these comparisons are presented in **Table 3** and **Figure 4**.

**Table 3:** Analysis of the variables BES, WBIS, and PWS in the SEX and BMI groups.

	BES		WBIS		PWS	
Cases	F	p	F	p	F	p
SEX	4.08	0.04	0.36	0.55	0.13	0.72
BMI_Groups	7.60	<.001	18.32	<.001	8.17	<.001
SEX ✻ BMI_Groups	2.17	0.12	9.73	<.001	0.59	0.55

**Figure 5:**
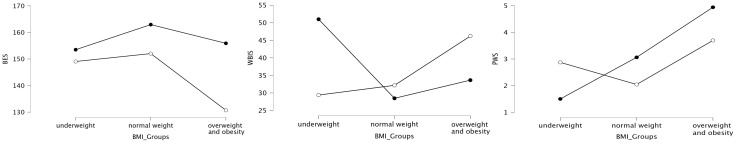
The graphical representation of the results comparing stress levels in the SEX and BMI groups.

The analyses showed that the average levels of both chronic and acute stress differed according to BMI group and sex, but these differences were not statistically significant. The results are presented in **Table 4** and **Figure 5**.

**Table 4:** Analysis of stress levels according to sex and BMI group.

	Chronic stress		Acute stress	
Cases	F	p	F	p
SEX	0.53	0.47	2.36	0.12
BMI_Groups	1.73	0.18	1.12	0.33
SEX ✻ BMI_Groups	0.30	0.74	0.17	0.84

**Figure 6:**
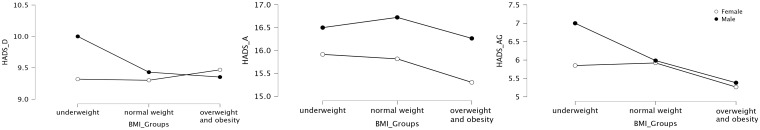
The graphical representation of the mean results comparing HADS scores according to SEX and BMI groups.

The analysis of the collected data with the HADS questionnaire revealed differences only in the HADS_AG. The mean scores for aggression were lower in the overweight and obese group than in the normal weight group (_Ptukey_<0.01). The results are shown in **Table 5** and **Figure 6**.

**Table 5:** Analysis of HADS scores according to sex and BMI group.

	HADS_D		HADS_A		HADS_AG	
Cases	F	p	F	p	F	p
SEX	0.20	0.65	1.56	0.21	1.80	0.18
BMI_Groups	0.08	0.92	0.95	0.39	7.47	<.001
SEX ✻ BMI_Groups	0.20	0.82	0.02	0.98	0.65	0.52

The presented analyses indicate that controlled variables in the form of sex and BMI groups should be included in subsequent analyses.

The next step was to establish the relationships between the variables. For this purpose, a correlation analysis was performed. The results are presented in **Supplementary Table 1**. The analysis showed a statistically significant correlation between persistence and the number of tasks performed (r=0.47; p<0.001). A weaker association was shown between persistence and time spent on tasks (r=0.16; p<0.001). A negative relationship was also shown between acute stress level and persistence indices, as determined by The Maze Test. No relationship was observed between acute stress and persistence as a trait. The persistence indices were also shown to depend on temperamental personality factors to a greater extent than persistence, as measured by the PS-20 test. Depressive symptoms were negatively associated with the studied persistence factors, while anxiety and anger were positively associated.

Body image, as measured by the BES scale, was found to be associated with persistence regardless of how it was measured. There was a significant relationship between the strength of the BES and the persistence trait (r=0.46; p<0.001) as well as between the strength of the BES and the number of tasks performed (r=0.48; p<0.001). The association between BES and time spent on tasks was also significant, but the strength of the association was low. There was also a negative relationship between the medium-strength BES and the PWS (r=-0.46; p<0.001) as well as a negative association between the low-strength BES and the WBIS (r=-0.27; p<0.001). PWS was also negatively associated with persistence_trait (r=-0.64; p<0.001) and number of tasks performed (r=-0.41; p<0.001). On the other hand, WBIS was negatively associated with the number of tasks performed (r=-0.14; p<0.001) and time spent on tasks (r=-0.28; p<0.001).

The analyses showed a relationship between persistence and body image as well as between weight-related stereotypes and stigmas. These associations are characterized by medium strength correlations. Persistence, regardless of approach, is also related to personal-temperamental characteristics.

Comparisons were also made between the strength of correlations of body image with persistence and variables describing the internalization of stereotypes and perceived stigma as well as with regard to the controlled variables of sex and normality of body weight. The association of PWS with BES was shown to be significantly (p<0.01) stronger in the female group (r=-0.56) than in the male group (r=-0.31). The relationship between BES and persistence is strongest in the underweight group. A comparison of the association between BES and persistence in the normal weight, overweight, and obese groups revealed significant differences only in time spent on tasks (p=0.01). A difference was also shown at the trend level with the number of tasks performed (p=0.06). In contrast, the relationship between the BES and the WBIS was found to be strongest in the normal weight group. The association of the PWS with BES was strongest in the underweight group (r=-0.82) and second-strongest in the overweight and obese group (r=-0.41). It was the weakest in the normal weight group. The results of these comparisons are presented in **Table 6**.

**Table 6:** Comparison of correlations in the SEX and BMI groups.

Correlation BES	SEX		p Fisher Z Test	BMI			p Fisher Z Test		
	Female	Male		to low (1)	Normal (2)	to high (3)	1-2	1-3	2-3
Persistence (PS-20)	0.50 ***	0.40 ***	0.09	0.72 ***	0.39 ***	0.42 ***	<0.01	<0.01	0.33
Number of tasks performed	0.49 ***	0.45 ***	0.30	0.80 ***	0.36 ***	0.47 ***	<0.01	<0.01	0.06
Time spent on tasks	0.17 ***	0.17 *	0.50	0.52 ***	0.05	0.23 ***	<0.01	<0.01	0.01
WBIS	-0.28 ***	-0.20 *	0.18	-0.04	-0.37 ***	-0.16 *	<0.01	0.16	<0.01
PWS	-0.56 ***	-0.31 ***	<0.01	-0.82 ***	-0.21 ***	-0.41 ***	<0.01	<0.01	<0.01

*p <.05, **p<.01, ***p <.001.

**Figure 7:**
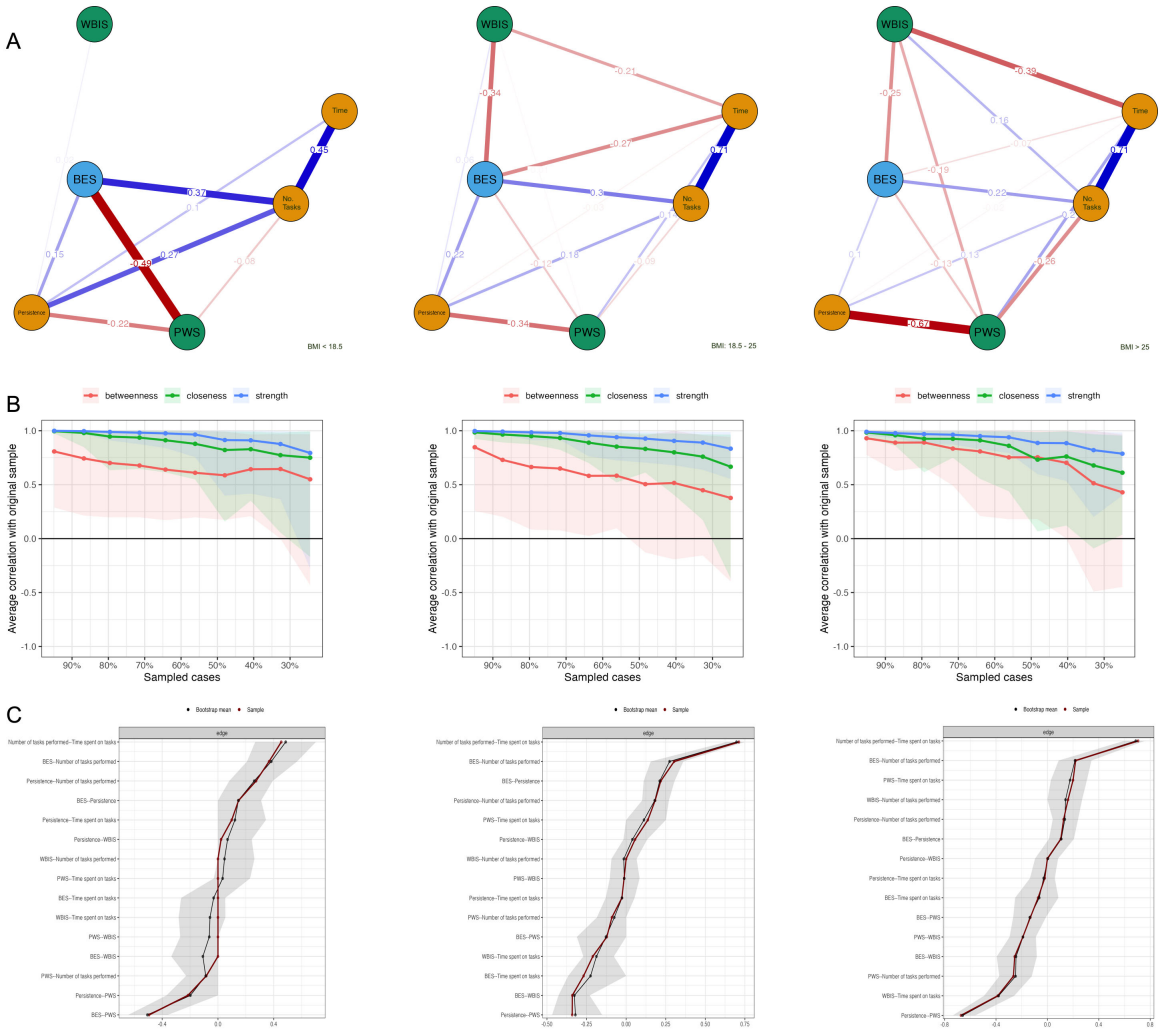
SEX-disaggregated network model **(A)**. Average correlations between centrality indices of networks sampled with persons dropped and the original sample **(B)**. *Lines* indicate the means and *areas* indicate the range from the 2.5th quantile to the 97.5th quantile. Bootstrapped confidence intervals of estimated edge-weights for the estimated network **(C)**. The red line indicates the sample values and the gray area the bootstrapped CIs.

**Figure 8:**
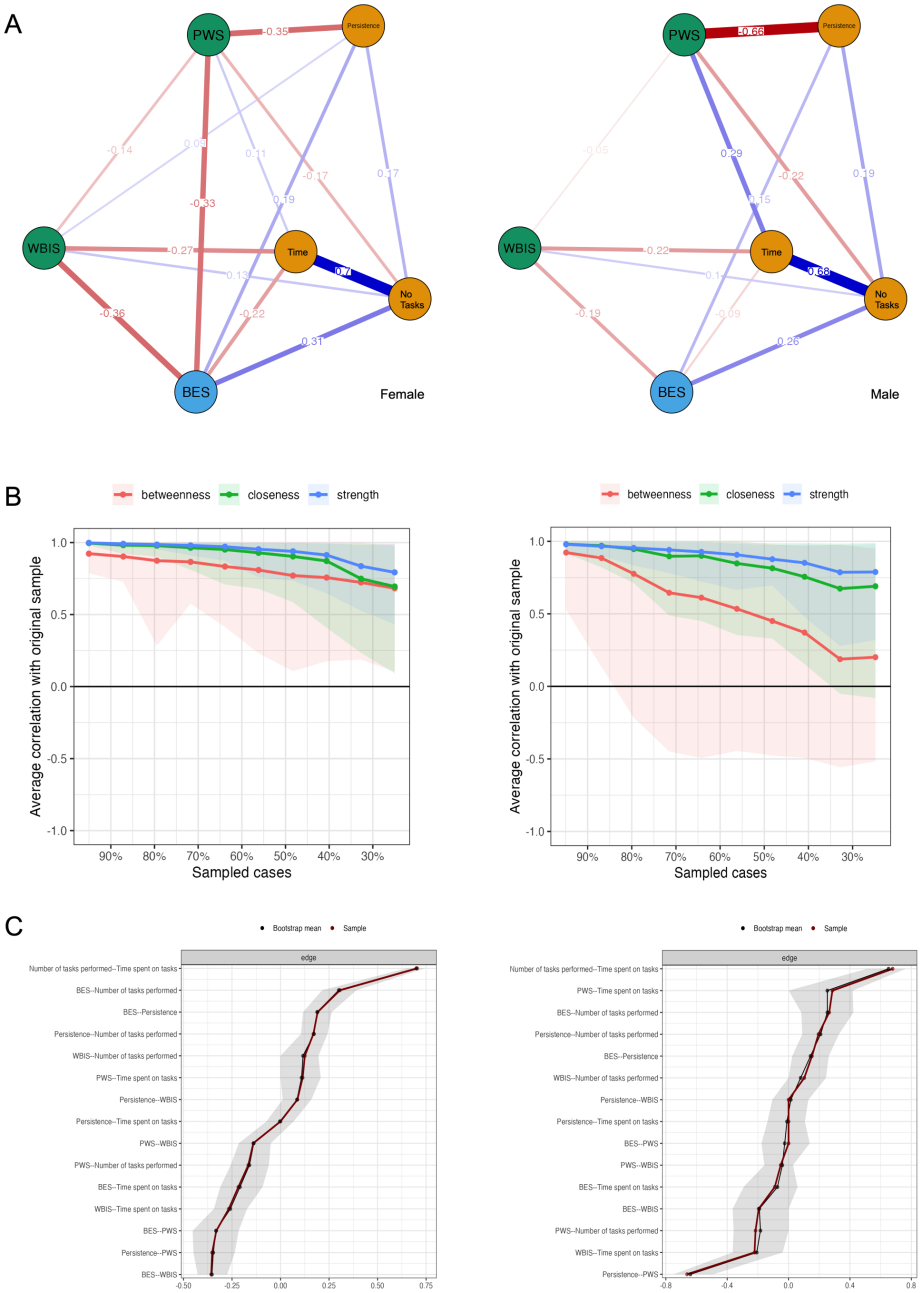
Network model subdivided by BMI group **(A)**. Average correlations between centrality indices of networks sampled with persons dropped and the original sample **(B)**. *Lines* indicate the means and *areas* indicate the range from the 2.5th quantile to the 97.5th quantile. Bootstrapped confidence intervals of estimated edge-weights for the estimated network **(C)**. The red line indicates the sample values and the gray area the bootstrapped CIs.

To confirm the obtained results and graphically depict the relationships between the main variables in this study, a network analysis was conducted. The estimated network models contain variables (nodes) and estimated links between them (edges). The analysis of network stability indicates that the proximity between nodes is not stable. Node strength and closeness performs better and can be interpreted with caution. The stability and accuracy of the network are shown in **Figures 7** and **8**. Two models were created for comparison; the first is presented in **Figure 7**, with separate groups stratified by sex. A comparison of the models shows that the BES linkage of variables appears in the female group. The edges connecting the BES node to the PWS, WBIS, and Time are between -0.22 and -0.36. These links are weaker in the male group, however, in contrast to the female group, a stronger association of PWS with Persistence_Trait and PWS with Time was observed. The association of BES with NoTasks was weaker. The second model included separate BMI groups and is presented in **Figure 8**. This model shows an increase in the association of WBIS with the study variables as BMI increases. The model also showed the strongest PWS-BES-NoTasks-Persistence associations in the underweight group. In the overweight and obese group, the strongest associations were observed between the nodes Persistence - PWS and WBIS - Time persistence (PWS), and WBIS time points.

## Discussion

Persistence is a construct that has rarely been analyzed in the context of its association with body image, especially in relation to the normality of one’s body weight. The main aim of our study was to replicate this study by presenting preliminary results focused on the relationship between body image and persistence. We additionally searched for answers to questions about the relationships between the studied associations and body weight stereotypes.

In accordance with previous research, our study confirmed that women rate their bodies less favorably than men do and this effect has been observed over the years (69–71). Women pay particular attention to the assessment of their face and figure (72) and there is clear pressure to meet strict standards of appearance, making body and beauty key categories for women’s evaluation. Therefore, the link between body image and the experience of being stereotyped and prejudiced was found to be stronger for women than for men.

Stigma and stigmatization toward appearance are formulated not only against excessive body weight but also against people with an atypical body shape in general, which can significantly affect their body image. A number of studies indicate that dissimilarity from the contemporary standard of beauty in terms of body weight is stigmatized in both overly slim and overly fat figures (73). This effect was also confirmed in our study, which showed that respondents’ body image significantly depends on body weight. We know from empirical reports that negative experiences of self-perception can affect people’s ability to cope with life difficulties. Dealing with excessive body weight is a demanding, long-term effort. Unfortunately, being overweight is related to prejudice and the daily experience of various problems associated with it. Trying to control one’s diet requires persistence, but stigma can result in uncontrollable overeating and less interest in physical activity (74, 75). In the academic literature, there is less interest in the stigmatization of thin people. This may be due to the focus on the obesity epidemic. However, our findings suggest that the problematic weight experiences of thin people should receive similar attention.

The study also revealed that respondents’ BMI, rather than sex, was associated with the internalization of stereotypes. Moreno-Domingues (76) suggested that BMI may not only be responsible for poorer self-image but also may moderate the relationship between the internalization of the beauty ideal and self-image. Our study revealed an association between BMI and the internalization of weight stereotypes as well as between BMI and the level of experience of stereotypes and prejudice. We believe that people with a higher BMI internalize cultural stereotypes more strongly and that the perceived difference between their own appearance and the promoted body shape matters for self-image. At the same time, they experience more negative behaviors that stigmatize their body weight. The acceptance of social judgments regarding obesity exacerbates the negative motivational effects of obesity stigma (77). Consequently, it can inhibit actions aimed at coping with excessive body weight. It is also fostered by the depletion of resources needed to modify one’s weight and is used to cope with the stigma of obesity in the long term (78).

The results on persistence demonstrate that it is not dependent on either the sex or BMI of the respondents. The level of persistence was significantly associated with body perception, confirming previous findings by Styk et al. (13) The current study revealed an association between persistence and the experience of weight stigma, particularly in individuals with below-normal body weight and overweight participants. It is known from other studies that body nonconformity with beauty can be significantly stigmatized (29, 50, 79). Obese individuals experience dehumanization that fosters prejudice (80). In the case of nonnormal-weight individuals, we can expect weaker persistence; thus, oppressive social messages about weight may more strongly affect their evaluation of their body. Based on the literature review and our study, it is possible to speculate that this process may sabotage any attempts at weight modification; however, this requires further research.

In summary, among the studied variables (temperament, personality, body image, experience of stereotypes and prejudices, and internalization of stereotypes), only body stigma is socially controlled. The obtained results may indicate a self-sustaining mechanism that makes it difficult for overweight or obese people to correct their weight. As indicated in the original study<sup>53</sup>, deficits in persistence can affect, among other things, problems in maintaining a proper and healthy diet, which in turn can increase body weight and lead to being overweight and obese. Obesity and being overweight, in turn, can build up a negative body image. Therefore, it cannot be ruled out that overweight and obese individuals may experience a kind of feedback loop that increases persistence deficits along with negative body image. Consequently, these persistence deficits may lead to problems maintaining one’s diet, which may result in weight gain and further exacerbation of negative body image. In the current study, the strength by which this loop is coupled is likely to be influenced by existing weight stereotypes, as the results indicate. A new and extremely interesting result was the demonstrated relationship in the underweight group. The association between body image and the experience of weight stigma was strongest in this group. The second strongest relationship was between body image and persistence. This indicates that body-related stigma not only affects overweight and obese individuals but also that its mechanisms may be shaped differently.

We should, therefore, seek interventions to break this loop and unleash the potential in obese people to persistently and effectively manage their excess body weight. By investing in the de-stereotyping of obese people, prejudice reduction and inclusion activities can support a positive self-image in this group.

Like any study, this one has certain limitations. First, it included people only up to the age of 30, so it is not possible to generalize the results to the entire population. A smaller group of men results in a lower possibility of drawing conclusions, especially for underweight men. Future studies should focus on this group and include factors related to social media and the ultraslim female and supermuscular male physiques promoted in the media.

## Conclusion and implications

Body image and persistence are related variables. They are associated with the internalization of stereotypes and perceived stigma related to body weight. These associations are differentially shaped according to sex and the regularity of body weight. In women, a stronger association of these variables with body image was observed, while in men, the relationship with body image was weaker, with a stronger association shown by perceived weight-related stigma. In the group of participants with a BMI<18.5, there was no significant association between the internalization of stereotypes and the analyzed variables. This relationship appeared in the group of subjects with a normal body weight and was strongest in the group of participants who were overweight or obese. Perceived weight-related stigma was most strongly associated with body image in the group with BMI<18.5 kg/m2 and with persistence in the group with BMI>25 kg/m2.

Public education on the complex determinants of not only obesity but also body weight in general is key to reducing ‘weight stigma’. The implications of this study should include actions to eliminate stigmatizing language in public spaces, especially in educational settings, the healthcare system, and the media. In addition to the medical aspects, the psychological consequences of prejudice and discrimination should be addressed, and positive bodily content and empathetic attitudes should be promoted to reduce harmful behavior.

## Data Availability

The raw data supporting the conclusions of this article will be made available by the authors, without undue reservation.
