# Corrigendum: Body image is associated with persistence. A study of the role of weight-related stigma

**DOI:** 10.3389/fpsyt.2025.1591058

**Published:** 2025-04-01

**Authors:** Wojciech Styk, Ewa Wojtowicz, Paweł Glibowski, Katarzyna Iłowiecka, Aleksanda Jędryszek-Geisler, Szymon Zmorzyński

**Affiliations:** ^1^ Academic Laboratory of Psychological Tests, Medical University of Lublin, Lublin, Poland; ^2^ Chair of Pedeutology and Psychology of Education, Christian Theological Academy of Warsaw, Warsaw, Poland; ^3^ Department of Biotechnology, Microbiology and Human Nutrition, Faculty of Food Sciences and Biotechnology, University of Life Sciences in Lublin, Lublin, Poland; ^4^ Nutrition Clinic, Department of Clinical Dietetics Medical University of Lublin, Lublin, Poland; ^5^ Department of Psychology, Institute of Pedagogy and Psychology, Management Academy of Applied Sciences in Warsaw, Warsaw, Poland; ^6^ Laboratory of Genetics, Academy of Zamość, Zamość, Poland

**Keywords:** persistence, body image, body mass index, weight stigma, stress, cortisol, dehydroepiandrosterone, temperament

In the published article, there was an error in affiliation 4. Instead of “Department of Food and Nutrition, Medical University of Lublin, Lublin, Poland”, it should be “Nutrition Clinic, Department of Clinical Dietetics Medical University of Lublin, Lublin, Poland”.

The authors apologize for this error and state that this does not change the scientific conclusions of the article in any way. The original article has been updated.

