# Supplementary material for: Body image is associated with persistence. A study of the role of weight-related stigma
Source: Front Psychiatry. 2024 Oct 25;15:1464939. doi: 10.3389/fpsyt.2024.1464939 (PMC11549672; doi:10.3389/fpsyt.2024.1464939)

SUPPLEMENTARY

PWS

Poniżej znajduje się lista stwierdzeń dotyczących interakcji z innymi osobami. Jeżeli dana sytuacja miała miejsce z powodu Twojej masy ciała zakteśl TAK, w przeciwnym razie zakreśl NIE

| 1. Ludzie zachowują się jakbyś był gorszy | TAK/NIE |
| --- | --- |
| 1. Ludzie zachowują się jak byś nie był inteligentny | TAK/NIE |
| 1. Ludzie zachowują się tak, jakby się Ciebie bali | TAK/NIE |
| 1. Jesteś traktowany z mniejszą uprzejmością niż inni | TAK/NIE |
| 1. Jesteś traktowany z mniejszym szacunkiem niż inni | TAK/NIE |
| 1. Otrzymujesz kiepskie usługi w sklepach/restauracjach | TAK/NIE |
| 1. Ludzie zachowują się jak byś był nieuczciwy | TAK/NIE |
| 1. Jesteś wyzywany lub obrażany | TAK/NIE |
| 1. Jesteś zastraszany lub nękany | TAK/NIE |
| 1. Spotkałeś się z dyskryminacją w wielu środowiskach społecznych np. w szkole czy pracy | TAK/NIE |

WBIS

Na skali 1-7, gdzie 1 oznacza "w pełni się nie zgadzam” a 7 oznacza "w pełni się zgadzam”

określ na ile zgadzasz się z podanymi zdaniami.

| 1. Z powodu mojej masy ciała czuję, że jestem tak samo kompetentny jak każdy. | 1 2 3 4 5 6 7 |
| --- | --- |
| 1. Z powodu mojej masy ciała jestem mniej atrakcyjna niż większość innych ludzi. | 1 2 3 4 5 6 7 |
| 1. Czuję niepokój związany z moją masą ciała z powodu tego, co ludzie mogą o mnie pomyśleć. | 1 2 3 4 5 6 7 |
| 1. Chciałabym radykalnie zmienić swoją masę ciała. | 1 2 3 4 5 6 7 |
| 1. Zawsze, gdy dużo myślę o swojej masie, czuję się przygnębiona. | 1 2 3 4 5 6 7 |
| 1. Nienawidzę siebie za moją masę ciała. | 1 2 3 4 5 6 7 |
| 1. Moja masa ciała jest głównym sposobem, w jaki oceniam swoją wartość jako osoby. | 1 2 3 4 5 6 7 |
| 1. Nie czuję, że z powodu mojej masy ciała zasługuję na naprawdę satysfakcjonujące życie towarzyskie. | 1 2 3 4 5 6 7 |
| 1. Dobrze się czuję będąc w takiej masie ciała, w jakiej jestem. | 1 2 3 4 5 6 7 |
| 1. Z powodu mojej masy ciała nie czuję się jak moje prawdziwe ja. | 1 2 3 4 5 6 7 |
| 1. Z powodu mojej masy nie rozumiem, jak ktoś atrakcyjny może chcieć się ze mną umówić. | 1 2 3 4 5 6 7 |

**Descriptive statistics**

| **Descriptives - Persistence** | | | | | | | | | | | | |
| --- | --- | --- | --- | --- | --- | --- | --- | --- | --- | --- | --- | --- |
| **SEX** | | **BMI_Groups** | | **N** | | **Mean** | | **SD** | | **SE** | |  |
| Female |  | normal weight |  | 330 |  | 87.41 |  | 23.72 |  | 1.31 |  |  |
|  |  | overweight and obesity |  | 160 |  | 85.23 |  | 28.57 |  | 2.27 |  |  |
|  |  | underweight |  | 94 |  | 91.32 |  | 27.47 |  | 2.83 |  |  |
| Male |  | normal weight |  | 65 |  | 84.78 |  | 28.66 |  | 3.55 |  |  |
|  |  | overweight and obesity |  | 68 |  | 81.15 |  | 37.51 |  | 4.55 |  |  |
|  |  | underweight |  | 4 |  | 92.50 |  | 22.52 |  | 11.26 |  |  |
|  | | | | | | | | | | | | |

| **Descriptives - Number of tasks performed** | | | | | | | | | | | | |
| --- | --- | --- | --- | --- | --- | --- | --- | --- | --- | --- | --- | --- |
| **SEX** | | **BMI_Groups** | | **N** | | **Mean** | | **SD** | | **SE** | |  |
| Female |  | normal weight |  | 330 |  | 191.84 |  | 38.35 |  | 2.11 |  |  |
|  |  | overweight and obesity |  | 160 |  | 187.59 |  | 49.95 |  | 3.95 |  |  |
|  |  | underweight |  | 94 |  | 184.49 |  | 57.96 |  | 5.98 |  |  |
| Male |  | normal weight |  | 65 |  | 200.09 |  | 37.95 |  | 4.71 |  |  |
|  |  | overweight and obesity |  | 68 |  | 204.01 |  | 62.68 |  | 7.60 |  |  |
|  |  | underweight |  | 4 |  | 200.00 |  | 23.09 |  | 11.55 |  |  |
|  | | | | | | | | | | | | |

| **Descriptives - Time spent on tasks** | | | | | | | | | | | | |
| --- | --- | --- | --- | --- | --- | --- | --- | --- | --- | --- | --- | --- |
| **SEX** | | **BMI_Groups** | | **N** | | **Mean** | | **SD** | | **SE** | |  |
| Female |  | normal weight |  | 330 |  | 106.83 |  | 39.79 |  | 2.56 |  |  |
|  |  | overweight and obesity |  | 160 |  | 105.16 |  | 37.46 |  | 2.82 |  |  |
|  |  | underweight |  | 94 |  | 106.79 |  | 43.95 |  | 4.55 |  |  |
| Male |  | normal weight |  | 65 |  | 117.23 |  | 38.63 |  | 4.06 |  |  |
|  |  | overweight and obesity |  | 68 |  | 129.12 |  | 50.43 |  | 6.65 |  |  |
|  |  | underweight |  | 4 |  | 140.00 |  | 30.34 |  | 15.67 |  |  |
|  | | | | | | | | | | | | |

| **Descriptives - BES** | | | | | | | | | | | | |
| --- | --- | --- | --- | --- | --- | --- | --- | --- | --- | --- | --- | --- |
| **SEX** | | **BMI_Groups** | | **N** | | **Mean** | | **SD** | | **SE** | |  |
| Female |  | normal weight |  | 330 |  | 152.03 |  | 29.68 |  | 1.64 |  |  |
|  |  | overweight and obesity |  | 160 |  | 130.68 |  | 36.21 |  | 2.88 |  |  |
|  |  | underweight |  | 94 |  | 149.06 |  | 47.21 |  | 4.87 |  |  |
| Male |  | normal weight |  | 65 |  | 162.95 |  | 37.59 |  | 4.66 |  |  |
|  |  | overweight and obesity |  | 68 |  | 155.91 |  | 49.45 |  | 6.00 |  |  |
|  |  | underweight |  | 4 |  | 153.50 |  | 9.81 |  | 4.91 |  |  |
|  | | | | | | | | | | | | |

| **Descriptives - WBIS** | | | | | | | | | | | | |
| --- | --- | --- | --- | --- | --- | --- | --- | --- | --- | --- | --- | --- |
| **SEX** | | **BMI_Groups** | | **N** | | **Mean** | | **SD** | | **SE** | |  |
| Female |  | normal weight |  | 330 |  | 32.17 |  | 16.14 |  | 0.89 |  |  |
|  |  | overweight and obesity |  | 160 |  | 46.20 |  | 17.06 |  | 1.36 |  |  |
|  |  | underweight |  | 94 |  | 29.40 |  | 16.15 |  | 1.67 |  |  |
| Male |  | normal weight |  | 65 |  | 28.48 |  | 15.10 |  | 1.87 |  |  |
|  |  | overweight and obesity |  | 68 |  | 33.65 |  | 17.10 |  | 2.07 |  |  |
|  |  | underweight |  | 4 |  | 51.00 |  | 10.39 |  | 5.20 |  |  |
|  | | | | | | | | | | | | |

| **Descriptives - PWS** | | | | | | | | | | | | |
| --- | --- | --- | --- | --- | --- | --- | --- | --- | --- | --- | --- | --- |
| **SEX** | | **BMI_Groups** | | **N** | | **Mean** | | **SD** | | **SE** | |  |
| Female |  | normal weight |  | 330 |  | 2.04 |  | 3.29 |  | 0.18 |  |  |
|  |  | overweight and obesity |  | 160 |  | 3.70 |  | 5.17 |  | 0.41 |  |  |
|  |  | underweight |  | 94 |  | 2.87 |  | 4.91 |  | 0.51 |  |  |
| Male |  | normal weight |  | 65 |  | 3.06 |  | 4.94 |  | 0.61 |  |  |
|  |  | overweight and obesity |  | 68 |  | 4.94 |  | 6.83 |  | 0.83 |  |  |
|  |  | underweight |  | 4 |  | 1.50 |  | 0.58 |  | 0.29 |  |  |
|  | | | | | | | | | | | | |

| **Descriptives - Chronic stress** | | | | | | | | | | | | |
| --- | --- | --- | --- | --- | --- | --- | --- | --- | --- | --- | --- | --- |
| **SEX** | | **BMI_Groups** | | **N** | | **Mean** | | **SD** | | **SE** | |  |
| Female |  | normal weight |  | 330 |  | 4.72 |  | 1.71 |  | 0.09 |  |  |
|  |  | overweight and obesity |  | 160 |  | 4.82 |  | 1.85 |  | 0.15 |  |  |
|  |  | underweight |  | 94 |  | 4.13 |  | 1.55 |  | 0.16 |  |  |
| Male |  | normal weight |  | 65 |  | 4.31 |  | 1.63 |  | 0.20 |  |  |
|  |  | overweight and obesity |  | 68 |  | 4.68 |  | 1.69 |  | 0.20 |  |  |
|  |  | underweight |  | 4 |  | 4.00 |  | 1.15 |  | 0.58 |  |  |
|  | | | | | | | | | | | | |

| **Descriptives - Acute stress** | | | | | | | | | | | | |
| --- | --- | --- | --- | --- | --- | --- | --- | --- | --- | --- | --- | --- |
| **SEX** | | **BMI_Groups** | | **N** | | **Mean** | | **SD** | | **SE** | |  |
| Female |  | normal weight |  | 330 |  | 2.97 |  | 1.85 |  | 0.10 |  |  |
|  |  | overweight and obesity |  | 160 |  | 3.24 |  | 1.91 |  | 0.15 |  |  |
|  |  | underweight |  | 94 |  | 2.76 |  | 1.91 |  | 0.20 |  |  |
| Male |  | normal weight |  | 65 |  | 2.66 |  | 1.64 |  | 0.20 |  |  |
|  |  | overweight and obesity |  | 68 |  | 2.76 |  | 1.75 |  | 0.21 |  |  |
|  |  | underweight |  | 4 |  | 2.00 |  | 0.00 |  | 0.00 |  |  |
|  | | | | | | | | | | | | |

| **Descriptives - HADS_D** | | | | | | | | | | | | |
| --- | --- | --- | --- | --- | --- | --- | --- | --- | --- | --- | --- | --- |
| **SEX** | | **BMI_Groups** | | **N** | | **Mean** | | **SD** | | **SE** | |  |
| Female |  | normal weight |  | 330 |  | 9.30 |  | 2.88 |  | 0.16 |  |  |
|  |  | overweight and obesity |  | 160 |  | 9.47 |  | 2.98 |  | 0.24 |  |  |
|  |  | underweight |  | 94 |  | 9.32 |  | 2.19 |  | 0.23 |  |  |
| Male |  | normal weight |  | 65 |  | 9.43 |  | 2.73 |  | 0.34 |  |  |
|  |  | overweight and obesity |  | 68 |  | 9.35 |  | 3.17 |  | 0.38 |  |  |
|  |  | underweight |  | 4 |  | 10.00 |  | 0.00 |  | 0.00 |  |  |
|  | | | | | | | | | | | | |

| **Descriptives - HADS_A** | | | | | | | | | | | | |
| --- | --- | --- | --- | --- | --- | --- | --- | --- | --- | --- | --- | --- |
| **SEX** | | **BMI_Groups** | | **N** | | **Mean** | | **SD** | | **SE** | |  |
| Female |  | normal weight |  | 330 |  | 15.82 |  | 3.27 |  | 0.18 |  |  |
|  |  | overweight and obesity |  | 160 |  | 15.30 |  | 3.86 |  | 0.31 |  |  |
|  |  | underweight |  | 94 |  | 15.91 |  | 3.61 |  | 0.37 |  |  |
| Male |  | normal weight |  | 65 |  | 16.72 |  | 3.61 |  | 0.45 |  |  |
|  |  | overweight and obesity |  | 68 |  | 16.26 |  | 4.24 |  | 0.51 |  |  |
|  |  | underweight |  | 4 |  | 16.50 |  | 4.04 |  | 2.02 |  |  |
|  | | | | | | | | | | | | |

| **Descriptives - HADS_AG** | | | | | | | | | | | | |
| --- | --- | --- | --- | --- | --- | --- | --- | --- | --- | --- | --- | --- |
| **SEX** | | **BMI_Groups** | | **N** | | **Mean** | | **SD** | | **SE** | |  |
| Female |  | normal weight |  | 330 |  | 5.92 |  | 1.46 |  | 0.08 |  |  |
|  |  | overweight and obesity |  | 160 |  | 5.27 |  | 2.04 |  | 0.16 |  |  |
|  |  | underweight |  | 94 |  | 5.85 |  | 2.03 |  | 0.21 |  |  |
| Male |  | normal weight |  | 65 |  | 5.98 |  | 1.78 |  | 0.22 |  |  |
|  |  | overweight and obesity |  | 68 |  | 5.38 |  | 2.41 |  | 0.29 |  |  |
|  |  | underweight |  | 4 |  | 7.00 |  | 0.00 |  | 0.00 |  |  |
|  | | | | | | | | | | | | |

## Network Analysis 1

1 – BMI<18.5

2 – BMI (18.5 – 25)

3 – BMI>25

| **Summary of Network** | | | | | | | |
| --- | --- | --- | --- | --- | --- | --- | --- |
| **Network** | | **Number of nodes** | | **Number of nonzero edges** | | **Sparsity** | |
| 1 |  | 6 |  | 9/15 |  | 0.40 |  |
| 2 |  | 6 |  | 14/15 |  | 0.07 |  |
| 3 |  | 6 |  | 14/15 |  | 0.07 |  |
|  | | | | | | | |

| **Centrality measures per variable** | | | | | | | | | | | | | | | | | | | | | | | | | |
| --- | --- | --- | --- | --- | --- | --- | --- | --- | --- | --- | --- | --- | --- | --- | --- | --- | --- | --- | --- | --- | --- | --- | --- | --- | --- |
|  | | **1** | | | | | | | | **2** | | | | | | | | **3** | | | | | | | |
| **Variable** | | **Betweenness** | | **Closeness** | | **Strength** | | **Expected influence** | | **Betweenness** | | **Closeness** | | **Strength** | | **Expected influence** | | **Betweenness** | | **Closeness** | | **Strength** | | **Expected influence** | |
| BES |  | 0.00 |  | 0.36 |  | 0.72 |  | -0.28 |  | 0.71 |  | 1.22 |  | 0.75 |  | -0.46 |  | -0.65 |  | -1.32 |  | -1.27 |  | 0.03 |  |
| Persistence |  | 0.79 |  | 0.46 |  | 0.10 |  | 0.23 |  | 1.57 |  | -0.08 |  | -0.55 |  | 0.04 |  | -0.65 |  | -1.02 |  | -0.81 |  | -0.41 |  |
| PWS |  | -0.79 |  | 0.34 |  | 0.19 |  | -1.62 |  | -1.00 |  | -1.45 |  | -0.93 |  | -0.82 |  | 1.94 |  | 0.14 |  | 0.95 |  | -1.22 |  |
| WBIS |  | -0.79 |  | -2.02 |  | -1.73 |  | -0.28 |  | -1.00 |  | -0.84 |  | -1.18 |  | -0.95 |  | -0.65 |  | 0.29 |  | -0.58 |  | -0.69 |  |
| Number of tasks performed |  | 1.58 |  | 0.65 |  | 1.14 |  | 1.35 |  | -0.14 |  | 0.49 |  | 0.86 |  | 1.73 |  | 0.00 |  | 1.34 |  | 1.00 |  | 1.50 |  |
| Time spent on tasks |  | -0.79 |  | 0.21 |  | -0.41 |  | 0.60 |  | -0.14 |  | 0.66 |  | 1.06 |  | 0.46 |  | 0.00 |  | 0.56 |  | 0.70 |  | 0.79 |  |
|  | | | | | | | | | | | | | | | | | | | | | | | | | |

| **Clustering measures per variable** | | | | | | | | | | | | | | | | | | | | | | | | | |
| --- | --- | --- | --- | --- | --- | --- | --- | --- | --- | --- | --- | --- | --- | --- | --- | --- | --- | --- | --- | --- | --- | --- | --- | --- | --- |
|  | | **1** | | | | | | | | **2** | | | | | | | | **3** | | | | | | | |
| **Variable** | | **Barrat** | | **Onnela** | | **WS** | | **Zhang** | | **Barrat** | | **Onnela** | | **WS** | | **Zhang** | | **Barrat** | | **Onnela** | | **WS** | | **Zhang** | |
| Number of tasks performed |  | -0.19 |  | -0.08 |  | -0.03 |  | -0.63 |  | 0.97 |  | 1.67 |  | 1.29 |  | -0.23 |  | 0.26 |  | 0.67 |  | -0.65 |  | -0.43 |  |
| Time spent on tasks |  | 0.77 |  | 0.76 |  | 0.78 |  | 1.13 |  | -1.47 |  | 0.03 |  | -0.65 |  | -0.87 |  | -0.16 |  | -0.27 |  | -0.65 |  | -0.22 |  |
| BES |  | 0.77 |  | 0.94 |  | 0.78 |  | -0.12 |  | -0.87 |  | 0.62 |  | -0.65 |  | -0.06 |  | -0.84 |  | -1.12 |  | -0.65 |  | 0.88 |  |
| Persistence |  | -0.38 |  | -0.69 |  | -0.67 |  | 0.66 |  | -0.07 |  | -0.64 |  | -0.65 |  | -1.09 |  | 1.06 |  | -0.96 |  | 1.29 |  | 0.14 |  |
| PWS |  | 0.77 |  | 0.68 |  | 0.78 |  | 0.56 |  | 0.45 |  | -0.78 |  | -0.65 |  | 0.63 |  | -1.39 |  | 0.18 |  | -0.65 |  | -1.58 |  |
| WBIS |  | -1.74 |  | -1.61 |  | -1.65 |  | -1.60 |  | 0.97 |  | -0.89 |  | 1.29 |  | 1.61 |  | 1.06 |  | 1.50 |  | 1.29 |  | 1.22 |  |
|  | | | | | | | | | | | | | | | | | | | | | | | | | |

| **Weights matrix** | | | | | | | | | | | | | | | | | | | | | | | | | | | | | | | | | | | | | |
| --- | --- | --- | --- | --- | --- | --- | --- | --- | --- | --- | --- | --- | --- | --- | --- | --- | --- | --- | --- | --- | --- | --- | --- | --- | --- | --- | --- | --- | --- | --- | --- | --- | --- | --- | --- | --- | --- |
|  | | **1** | | | | | | | | | | | | **2** | | | | | | | | | | | | **3** | | | | | | | | | | | |
| **Variable** | | **BES** | | **Persistence** | | **PWS** | | **WBIS** | | **Number of tasks performed** | | **Time spent on tasks** | | **BES** | | **Persistence** | | **PWS** | | **WBIS** | | **Number of tasks performed** | | **Time spent on tasks** | | **BES** | | **Persistence** | | **PWS** | | **WBIS** | | **Number of tasks performed** | | **Time spent on tasks** | |
| BES |  | 0.00 |  | 0.15 |  | -0.49 |  | 0.00 |  | 0.37 |  | 0.00 |  | 0.00 |  | 0.22 |  | -0.12 |  | -0.34 |  | 0.30 |  | -0.27 |  | 0.00 |  | 0.10 |  | -0.13 |  | -0.25 |  | 0.22 |  | -0.07 |  |
| Persistence |  | 0.15 |  | 0.00 |  | -0.22 |  | 0.02 |  | 0.27 |  | 0.10 |  | 0.22 |  | 0.00 |  | -0.34 |  | 0.06 |  | 0.18 |  | -0.03 |  | 0.10 |  | 0.00 |  | -0.67 |  | 0.00 |  | 0.13 |  | -0.02 |  |
| PWS |  | -0.49 |  | -0.22 |  | 0.00 |  | 0.00 |  | -0.08 |  | 0.00 |  | -0.12 |  | -0.34 |  | 0.00 |  | -0.01 |  | -0.09 |  | 0.14 |  | -0.13 |  | -0.67 |  | 0.00 |  | -0.19 |  | -0.26 |  | 0.20 |  |
| WBIS |  | 0.00 |  | 0.02 |  | 0.00 |  | 0.00 |  | 0.00 |  | 0.00 |  | -0.34 |  | 0.06 |  | -0.01 |  | 0.00 |  | 0.00 |  | -0.21 |  | -0.25 |  | 0.00 |  | -0.19 |  | 0.00 |  | 0.16 |  | -0.39 |  |
| Number of tasks performed |  | 0.37 |  | 0.27 |  | -0.08 |  | 0.00 |  | 0.00 |  | 0.45 |  | 0.30 |  | 0.18 |  | -0.09 |  | 0.00 |  | 0.00 |  | 0.71 |  | 0.22 |  | 0.13 |  | -0.26 |  | 0.16 |  | 0.00 |  | 0.71 |  |
| Time spent on tasks |  | 0.00 |  | 0.10 |  | 0.00 |  | 0.00 |  | 0.45 |  | 0.00 |  | -0.27 |  | -0.03 |  | 0.14 |  | -0.21 |  | 0.71 |  | 0.00 |  | -0.07 |  | -0.02 |  | 0.20 |  | -0.39 |  | 0.71 |  | 0.00 |  |
|  | | | | | | | | | | | | | | | | | | | | | | | | | | | | | | | | | | | | | |

### Centrality Plot


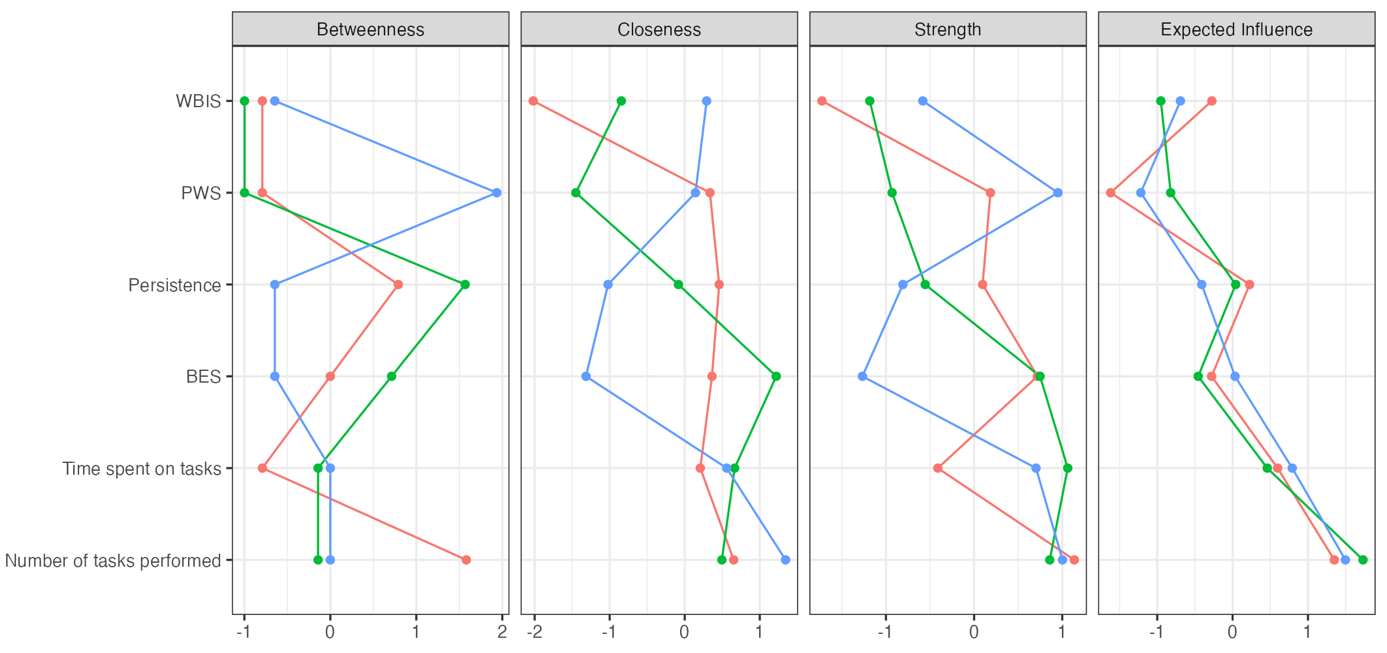

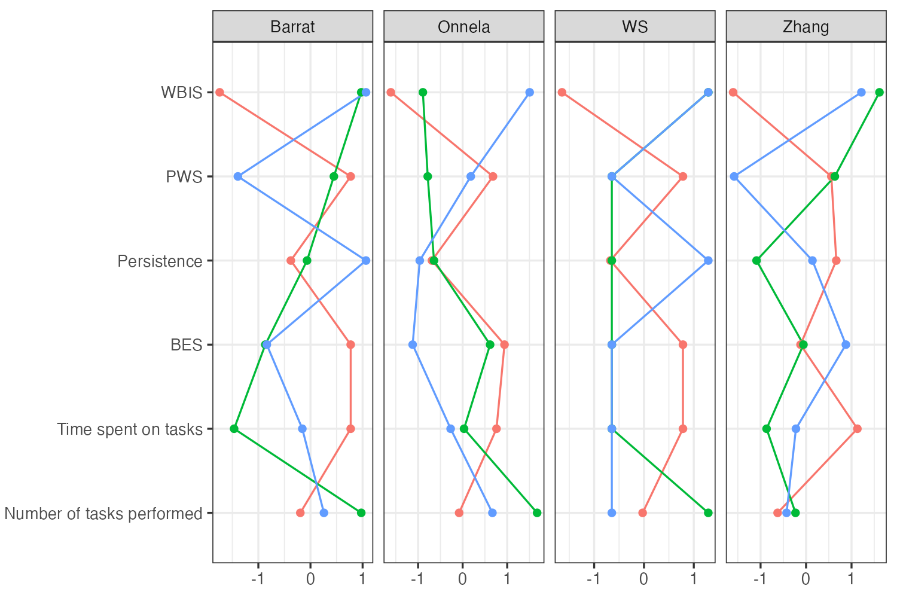


## Network Analysis 2

| **Summary of Network** | | | | | | | |
| --- | --- | --- | --- | --- | --- | --- | --- |
| **Network** | | **Number of nodes** | | **Number of nonzero edges** | | **Sparsity** | |
| Female |  | 6 |  | 14/15 |  | 0.07 |  |
| Male |  | 6 |  | 12/15 |  | 0.20 |  |
|  | | | | | | | |

| **Centrality measures per variable** | | | | | | | | | | | | | | | | | |
| --- | --- | --- | --- | --- | --- | --- | --- | --- | --- | --- | --- | --- | --- | --- | --- | --- | --- |
|  | | **Female** | | | | | | | | **Male** | | | | | | | |
| **Variable** | | **Betweenness** | | **Closeness** | | **Strength** | | **Expected influence** | | **Betweenness** | | **Closeness** | | **Strength** | | **Expected influence** | |
| BES |  | 1.19 |  | 1.66 |  | 0.87 |  | -0.50 |  | -1.00 |  | -0.63 |  | -0.97 |  | 0.08 |  |
| Persistence |  | -0.85 |  | -1.23 |  | -1.44 |  | 0.19 |  | -0.14 |  | -0.46 |  | -0.08 |  | -0.61 |  |
| PWS |  | -0.85 |  | -0.36 |  | -0.28 |  | -1.14 |  | 0.71 |  | -0.10 |  | 0.51 |  | -1.12 |  |
| WBIS |  | -0.85 |  | -0.46 |  | -0.76 |  | -0.70 |  | -1.00 |  | -1.20 |  | -1.36 |  | -0.69 |  |
| Number of tasks performed |  | 1.19 |  | 0.59 |  | 1.13 |  | 1.63 |  | -0.14 |  | 1.00 |  | 1.21 |  | 1.46 |  |
| Time spent on tasks |  | 0.17 |  | -0.20 |  | 0.48 |  | 0.52 |  | 1.57 |  | 1.39 |  | 0.69 |  | 0.88 |  |
|  | | | | | | | | | | | | | | | | | |

| **Clustering measures per variable** | | | | | | | | | | | | | | | | | |
| --- | --- | --- | --- | --- | --- | --- | --- | --- | --- | --- | --- | --- | --- | --- | --- | --- | --- |
|  | | **Female** | | | | | | | | **Male** | | | | | | | |
| **Variable** | | **Barrat** | | **Onnela** | | **WS** | | **Zhang** | | **Barrat** | | **Onnela** | | **WS** | | **Zhang** | |
| Number of tasks performed |  | -1.33 |  | -0.08 |  | -0.65 |  | -1.33 |  | -0.04 |  | 0.26 |  | -0.34 |  | -0.50 |  |
| Time spent on tasks |  | 1.17 |  | 1.67 |  | 1.29 |  | -0.12 |  | 1.28 |  | 1.59 |  | 1.28 |  | -0.46 |  |
| BES |  | -0.05 |  | 0.64 |  | -0.65 |  | -0.53 |  | -0.02 |  | -1.19 |  | -0.74 |  | 0.27 |  |
| Persistence |  | 1.17 |  | -0.51 |  | 1.29 |  | 0.81 |  | -0.90 |  | 0.46 |  | -0.74 |  | -0.90 |  |
| PWS |  | -0.61 |  | -0.69 |  | -0.65 |  | -0.31 |  | -1.28 |  | -0.30 |  | -0.74 |  | -0.30 |  |
| WBIS |  | -0.35 |  | -1.02 |  | -0.65 |  | 1.48 |  | 0.96 |  | -0.82 |  | 1.28 |  | 1.89 |  |
|  | | | | | | | | | | | | | | | | | |

| **Weights matrix** | | | | | | | | | | | | | | | | | | | | | | | | | |
| --- | --- | --- | --- | --- | --- | --- | --- | --- | --- | --- | --- | --- | --- | --- | --- | --- | --- | --- | --- | --- | --- | --- | --- | --- | --- |
|  | | **Female** | | | | | | | | | | | | **Male** | | | | | | | | | | | |
| **Variable** | | **BES** | | **Persistence** | | **PWS** | | **WBIS** | | **Number of tasks performed** | | **Time spent on tasks** | | **BES** | | **Persistence** | | **PWS** | | **WBIS** | | **Number of tasks performed** | | **Time spent on tasks** | |
| BES |  | 0.00 |  | 0.19 |  | -0.33 |  | -0.36 |  | 0.31 |  | -0.22 |  | 0.00 |  | 0.15 |  | 0.00 |  | -0.19 |  | 0.26 |  | -0.09 |  |
| Persistence |  | 0.19 |  | 0.00 |  | -0.35 |  | 0.09 |  | 0.17 |  | 0.00 |  | 0.15 |  | 0.00 |  | -0.66 |  | 0.00 |  | 0.19 |  | 0.00 |  |
| PWS |  | -0.33 |  | -0.35 |  | 0.00 |  | -0.14 |  | -0.17 |  | 0.11 |  | 0.00 |  | -0.66 |  | 0.00 |  | -0.05 |  | -0.22 |  | 0.29 |  |
| WBIS |  | -0.36 |  | 0.09 |  | -0.14 |  | 0.00 |  | 0.13 |  | -0.27 |  | -0.19 |  | 0.00 |  | -0.05 |  | 0.00 |  | 0.10 |  | -0.22 |  |
| Number of tasks performed |  | 0.31 |  | 0.17 |  | -0.17 |  | 0.13 |  | 0.00 |  | 0.70 |  | 0.26 |  | 0.19 |  | -0.22 |  | 0.10 |  | 0.00 |  | 0.68 |  |
| Time spent on tasks |  | -0.22 |  | 0.00 |  | 0.11 |  | -0.27 |  | 0.70 |  | 0.00 |  | -0.09 |  | 0.00 |  | 0.29 |  | -0.22 |  | 0.68 |  | 0.00 |  |
|  | | | | | | | | | | | | | | | | | | | | | | | | | |


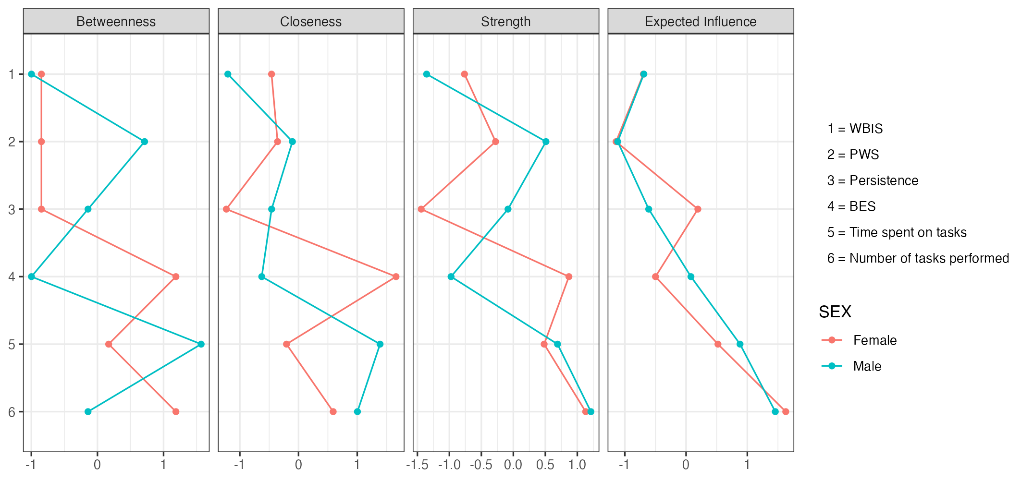


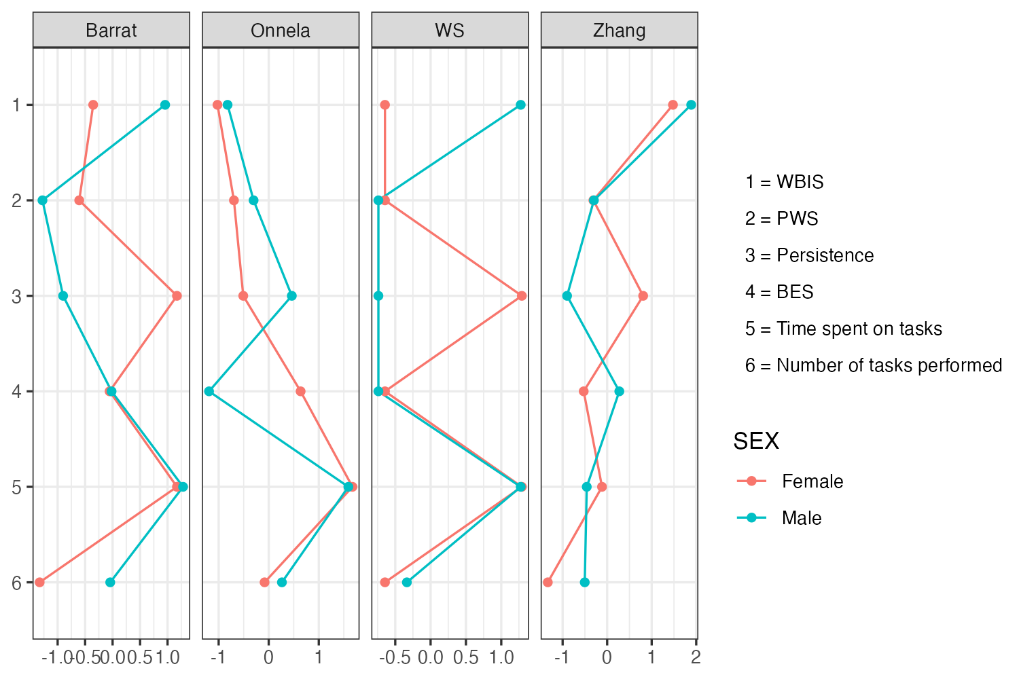

Supplement: Supplementary file 1 [file DataSheet1.docx]
