# Supplementary material for: Body image is associated with persistence. A study of the role of weight-related stigma
Source: Front Psychiatry. 2024 Oct 25;15:1464939. doi: 10.3389/fpsyt.2024.1464939 (PMC11549672; doi:10.3389/fpsyt.2024.1464939)
Supplement: Supplementary file 2 [file Table1.docx]

Supplementary Table 1. The results of the correlation analysis for the collected data

| **Variable** | | **1** | | **2** | | **3** | | **4** | | **5** | | **6** | | **7** | | **8** | | **9** | | **10** | | **11** | | **12** | | **13** | | **14** | | **15** | | **16** | | **17** | | **18** | | **19** | | **20** | | **21** | | **22** | | **23** | | **24** | | **25** | | |  |  |  |
| --- | --- | --- | --- | --- | --- | --- | --- | --- | --- | --- | --- | --- | --- | --- | --- | --- | --- | --- | --- | --- | --- | --- | --- | --- | --- | --- | --- | --- | --- | --- | --- | --- | --- | --- | --- | --- | --- | --- | --- | --- | --- | --- | --- | --- | --- | --- | --- | --- | --- | --- | --- | --- | --- | --- | --- |
| 1. Age |  | — |  |  |  |  |  |  |  |  |  |  |  |  |  |  |  |  |  |  |  |  |  |  |  |  |  |  |  |  |  |  |  |  |  |  |  |  |  |  |  |  |  |  |  |  |  |  |  |  | |  |  |  |  |
| 2. BMI |  | 0.11 | ** | — |  |  |  |  |  |  |  |  |  |  |  |  |  |  |  |  |  |  |  |  |  |  |  |  |  |  |  |  |  |  |  |  |  |  |  |  |  |  |  |  |  |  |  |  |  |  | |  |  |  |  |
| 3. Body fat (%) |  | 0.11 | ** | 0.73 | *** | — |  |  |  |  |  |  |  |  |  |  |  |  |  |  |  |  |  |  |  |  |  |  |  |  |  |  |  |  |  |  |  |  |  |  |  |  |  |  |  |  |  |  |  |  | |  |  |  |  |
| 4. Persistence (PS-20) |  | -0.01 |  | -0.11 | ** | -0.11 | ** | — |  |  |  |  |  |  |  |  |  |  |  |  |  |  |  |  |  |  |  |  |  |  |  |  |  |  |  |  |  |  |  |  |  |  |  |  |  |  |  |  |  |  | |  |  |  |  |
| 5. Number of tasks performed |  | 0.01 |  | 0.03 |  | -0.08 | * | 0.47 | *** | — |  |  |  |  |  |  |  |  |  |  |  |  |  |  |  |  |  |  |  |  |  |  |  |  |  |  |  |  |  |  |  |  |  |  |  |  |  |  |  |  | |  |  |  |  |
| 6. Time spent on tasks |  | 0.05 |  | 0.03 |  | -0.09 | * | 0.16 | *** | 0.69 | *** | — |  |  |  |  |  |  |  |  |  |  |  |  |  |  |  |  |  |  |  |  |  |  |  |  |  |  |  |  |  |  |  |  |  |  |  |  |  |  | |  |  |  |  |
| 7. BES |  | -0.11 | ** | -0.18 | *** | -0.31 | *** | 0.46 | *** | 0.48 | *** | 0.19 | *** | — |  |  |  |  |  |  |  |  |  |  |  |  |  |  |  |  |  |  |  |  |  |  |  |  |  |  |  |  |  |  |  |  |  |  |  |  | |  |  |  |  |
| 8. PWS |  | 0.08 | * | 0.17 | *** | 0.10 | ** | -0.64 | *** | -0.41 | *** | -0.04 |  | -0.46 | *** | — |  |  |  |  |  |  |  |  |  |  |  |  |  |  |  |  |  |  |  |  |  |  |  |  |  |  |  |  |  |  |  |  |  |  | |  |  |  |  |
| 9. WBIS |  | -0.08 | * | 0.29 | *** | 0.33 | *** | 0.02 |  | -0.14 | *** | -0.28 | *** | -0.27 | *** | -0.06 |  | — |  |  |  |  |  |  |  |  |  |  |  |  |  |  |  |  |  |  |  |  |  |  |  |  |  |  |  |  |  |  |  |  | |  |  |  |  |
| 10. HADS_AG |  | -0.10 | ** | -0.15 | *** | -0.14 | *** | 0.49 | *** | 0.37 | *** | 0.19 | *** | 0.38 | *** | -0.61 | *** | 0.03 |  | — |  |  |  |  |  |  |  |  |  |  |  |  |  |  |  |  |  |  |  |  |  |  |  |  |  |  |  |  |  |  | |  |  |  |  |
| 11. HADS_D |  | 0.03 |  | 0.04 |  | 0.02 |  | -0.22 | *** | -0.21 | *** | -0.22 | *** | -0.19 | *** | 0.13 | *** | 0.25 | *** | -0.16 | *** | — |  |  |  |  |  |  |  |  |  |  |  |  |  |  |  |  |  |  |  |  |  |  |  |  |  |  |  |  | |  |  |  |  |
| 12. HADS_A |  | 0.01 |  | -0.02 |  | -0.06 |  | 0.44 | *** | 0.52 | *** | 0.34 | *** | 0.42 | *** | -0.46 | *** | -0.12 | *** | 0.57 | *** | -0.40 | *** | — |  |  |  |  |  |  |  |  |  |  |  |  |  |  |  |  |  |  |  |  |  |  |  |  |  |  | |  |  |  |  |
| 13. Chronic stress |  | 0.13 | *** | 0.10 | ** | 0.06 |  | 0.01 |  | -0.08 | * | -0.09 | * | -0.05 |  | 0.03 |  | 0.10 | ** | -0.10 | ** | 0.08 | * | -0.20 | *** | — |  |  |  |  |  |  |  |  |  |  |  |  |  |  |  |  |  |  |  |  |  |  |  |  | |  |  |  |  |
| 14. Acute stress |  | 0.01 |  | 0.07 |  | 0.16 | *** | -0.10 |  | -0.22 | *** | -0.21 | *** | -0.17 | *** | 0.02 |  | 0.21 | *** | -0.12 | *** | 0.20 | *** | -0.31 | *** | 0.30 | *** | — |  |  |  |  |  |  |  |  |  |  |  |  |  |  |  |  |  |  |  |  |  |  | |  |  |  |  |
| 15. FCZKT_PE |  | -0.12 | ** | -0.06 |  | 0.05 |  | 0.05 |  | -0.22 | *** | -0.40 | *** | -0.02 |  | -0.10 | ** | 0.20 | *** | 0.01 |  | 0.13 | *** | -0.22 | *** | 0.12 | ** | 0.18 | *** | — |  |  |  |  |  |  |  |  |  |  |  |  |  |  |  |  |  |  |  |  | |  |  |  |  |
| 16. FCZKT_ER |  | 0.07 |  | -0.11 | ** | 0.04 |  | 0.06 |  | -0.24 | *** | -0.34 | *** | -0.01 |  | -0.14 | *** | 0.09 | * | -0.01 |  | 0.03 |  | -0.16 | *** | 0.09 | * | 0.12 | *** | 0.39 | *** | — |  |  |  |  |  |  |  |  |  |  |  |  |  |  |  |  |  |  | |  |  |  |  |
| 17. FCZKT_RH |  | 0.12 | ** | -0.02 |  | -0.09 | * | 0.14 | *** | 0.09 | * | 0.08 | * | 0.16 | *** | -0.03 |  | -0.13 | *** | 0.05 |  | -0.07 |  | 0.06 |  | 0.03 |  | -0.01 |  | 0.04 |  | 0.13 | *** | — |  |  |  |  |  |  |  |  |  |  |  |  |  |  |  |  | |  |  |  |  |
| 18. FCZKT_SS |  | 0.04 |  | 0.09 | * | 0.04 |  | 0.12 | *** | 0.09 | * | 0.03 |  | 0.06 |  | -0.03 |  | 0.03 |  | 0.01 |  | -0.11 | ** | 0.09 | * | 0.05 |  | 0.01 |  | 0.21 | *** | 0.07 |  | 0.03 |  | — |  |  |  |  |  |  |  |  |  |  |  |  |  |  | |  |  |  |  |
| 19. FCZKT_EN |  | 0.07 |  | 0.12 | *** | 0.04 |  | -0.14 | *** | 0.16 | *** | 0.28 | *** | -0.05 |  | 0.19 | *** | -0.06 |  | -0.08 | * | -0.06 |  | 0.02 |  | 0.05 |  | -0.07 | * | -0.36 | *** | -0.33 | *** | -0.07 |  | -0.06 |  | — |  |  |  |  |  |  |  |  |  |  |  |  | |  |  |  |  |
| 20. FCZKT_BR |  | 0.17 | *** | 0.16 | *** | 0.03 |  | 0.03 |  | 0.17 | *** | 0.28 | *** | -0.01 |  | 0.05 |  | -0.11 | ** | 0.01 |  | -0.13 | *** | 0.08 | * | 0.02 |  | -0.11 | ** | -0.28 | *** | -0.17 | *** | 0.03 |  | 0.10 | * | 0.24 | *** | — |  |  |  |  |  |  |  |  |  |  | |  |  |  |  |
| 21. FCZKT_AC |  | -0.05 |  | 0.04 |  | 0.04 |  | 0.17 | *** | 0.28 | *** | 0.09 | * | 0.26 | *** | -0.07 |  | -0.01 |  | 0.02 |  | -0.16 | *** | 0.13 | *** | -0.03 |  | -0.11 | ** | 0.06 |  | 0.07 |  | 0.15 | *** | 0.16 | *** | 0.02 |  | -0.09 | * | — |  |  |  |  |  |  |  |  | |  |  |  |  |
| 22. NEO_N |  | -0.12 | ** | -0.08 | * | 0.08 | * | -0.11 | ** | -0.47 | *** | -0.59 | *** | -0.15 | *** | -0.07 |  | 0.42 | *** | -0.03 |  | 0.32 | *** | -0.43 | *** | 0.14 | *** | 0.28 | *** | 0.50 | *** | 0.41 | *** | -0.12 | *** | -0.01 |  | -0.28 | *** | -0.32 | *** | -0.18 | *** | — |  |  |  |  |  |  | |  |  |  |  |
| 23. NEO_E |  | 0.06 |  | 0.04 |  | -0.04 |  | 0.31 | *** | 0.43 | *** | 0.12 | *** | 0.42 | *** | -0.20 | *** | -0.08 | * | 0.09 | * | -0.23 | *** | 0.25 | *** | -0.03 |  | -0.14 | *** | -0.10 | ** | -0.04 |  | 0.15 | *** | 0.11 | ** | 0.09 | * | 0.05 |  | 0.59 | *** | -0.25 | *** | — |  |  |  |  | |  |  |  |  |
| 24. NEO_O |  | 0.01 |  | -0.04 |  | -0.05 |  | 0.09 | * | 0.02 |  | 0.05 |  | -0.09 | * | 0.06 |  | 0.01 |  | -0.01 |  | -0.13 | *** | -0.08 | * | 0.04 |  | 0.03 |  | 0.16 | *** | 0.01 |  | -0.04 |  | 0.21 | *** | -0.03 |  | 0.01 |  | 0.10 | ** | 0.04 |  | 0.05 |  | — |  |  | |  |  |  |  |
| 25. NEO_A |  | 0.10 | ** | -0.15 | *** | -0.11 | ** | -0.07 |  | -0.16 | *** | 0.10 | ** | -0.18 | *** | 0.06 |  | -0.12 | ** | 0.04 |  | -0.09 | * | -0.06 |  | 0.01 |  | -0.01 |  | -0.02 |  | 0.18 | *** | -0.01 |  | 0.07 |  | 0.02 |  | 0.09 | * | -0.02 |  | -0.07 | * | 0.01 |  | 0.09 | * | — | |  |  |  |  |
| 26. NEO_C |  | 0.08 | * | -0.08 | * | -0.07 |  | 0.59 | *** | 0.35 | *** | 0.21 | *** | 0.41 | *** | -0.29 | *** | -0.08 | * | 0.29 | *** | -0.17 | *** | 0.27 | *** | 0.01 |  | -0.06 |  | -0.07 |  | 0.13 | *** | 0.36 | *** | 0.12 | ** | -0.01 |  | 0.20 | *** | 0.18 | *** | -0.19 | *** | 0.33 | *** | -0.02 |  | 0.07 | |  |  |  |  |
| * p < .05, ** p < .01, *** p < .001 |  |  |  |  |  |  |  |  |  |  |  |  |  |  |  |  |  |  |  |  |  |  |  |  |  |  |  |  |  |  |  |  |  |  |  |  |  |  |  |  |  |  |  |  |  |  |  |  |  | |  | |  |  |  |
